# Local Behavior of Lap-Spliced Deformed Rebars in Reinforced Concrete Beams

**DOI:** 10.3390/ma14237186

**Published:** 2021-11-25

**Authors:** Agha Syed Muhammad Gillani, Seung-Geon Lee, Soo-Hyung Lee, Hyerin Lee, Kee-Jeung Hong

**Affiliations:** School of Civil and Environmental Engineering, Kookmin University, Seoul 02707, Korea; aghasmg@gmail.com (A.S.M.G.); sug4682@kookmin.ac.kr (S.-G.L.); soohyung456@gmail.com (S.-H.L.)

**Keywords:** lap splice, bond, ductility, reinforced concrete beams, strain gauge, confinement, transverse reinforcement

## Abstract

Twelve full-scale reinforced concrete beams with two tension lap splices were constructed and tested under a four-point loading test. Half of these beams had shorter lap splices than that recommended by American Concrete Institute Building Code ACI 318-19; they failed by bond loss between steel and concrete at the lap splice region before rebar yielding. The other half of the beams were designed with a lap splice length slightly exceeding that recommended by ACI 318-19; they failed by rebar yielding and exhibited a ductile behavior. Several strain gauges were attached to the longitudinal bars in the lap splice region to study the local behavior of deformed bars during loading. The strain in a rebar was maximum at the loaded end of the lap splice and progressively decreased toward the unloaded end because the rebar at this end could not sustain any load. Stress flow discontinuity occurred at the loaded end and caused stress concentration. The effect of this concentration was investigated based on test results. The comparison of bond strengths calculated by existing equations and those of tested specimens indicated that the results agreed well.

## 1. Introduction

**Figure 1:**
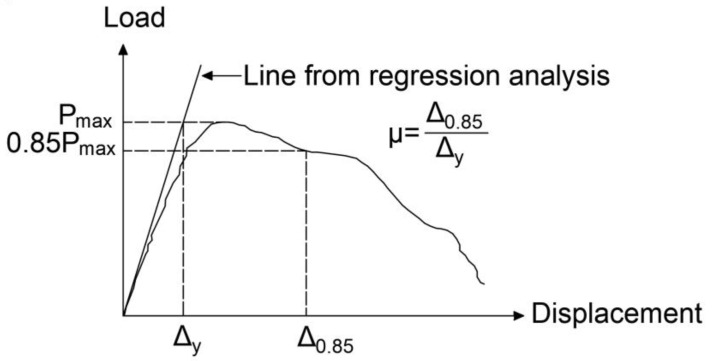
Definition of ductility.

The provision of lap splices is inevitable in reinforced concrete (RC) structures for several reasons: the unavailability of required rebar length on-site, the necessity of connecting rebars with different diameters, and the requisite of construction joints. Lap splices must be sufficiently long to provide adequate bond strength such that force can be transferred from one bar to the other. Moreover, the yielding of bars must occur before lap splice failure. Orangun et al. [1,2] performed a number of experiments on lap-spliced specimens and proposed an equation to predict the bond strength of lap splices. The equation specified by the ACI 318 (1995) [3] for a lap splice was also based on the equation developed by Orangun et al. [2]. Among the variables used in their formulated equation is the yield strength of transverse reinforcement. However, the strain gauge data of stirrups in lap-spliced specimens show that transverse reinforcement yielding is extremely rare, so it does not contribute to the bond strength of the lap splice [4,5,6,7,8,9]. Accordingly, more recent bond strength and development length equations do not include this factor. Azizinamini et al. [9] found that the equation specified by ACI 318 (1995) [3] does not ensure the ductile behavior of beams with tension lap splice. Moreover, a certain amount of transverse reinforcement must be provided over the lap splice to achieve sufficient ductility. The absence of transverse reinforcement causes splitting failure with concrete cover spalling in the lap splice region [10,11,12]. If transverse reinforcement is spaced apart outside the lap splice region, then splitting cracks can propagate toward this region; their widths can exceed those of cracks inside the lap splice region [13]. Cohn and Bartlett [14] defined ductility index, µ, as the ratio of displacement at 85% of the maximum load at the post-peak portion of the load–displacement curve to the displacement corresponding to the intersection of tangents (i.e., tangents to the load–displacement curve at the origin and point of maximum displacement), as shown in Figure 1 [14].

The geometric properties of bars also affect the lap splice strength. Hassan and Feldman [15] proposed an equation for predicting the maximum load resistance for plain lap-spliced bars based on their experimental study using 11 specimens. With their limited data, they found that the maximum load resistance of plain bars was approximately 60% of those recorded for identical specimens reinforced with deformed bars. Three other specimens were instrumented with concrete and steel strain gauges to study the bond stress distribution in the lap splice region. They observed that bond demand was initially greatest at the ends of the lap splice. Subsequently, bond loss occurred in these regions as the bond demand adjacent to the midpoint of the lap splice increased. Similar results were reported by Garcia et al. [16] who studied the strain behavior of lap splices by attaching seven strain gauges to one of the two lap splices provided in RC beams.

Wu et al. [17], Garcia et al. [18], and Gaurav and Singh [19] studied the strain behavior of lap-spliced bars by attaching steel strain gauges just outside the ends of the splice. Mousa [12] and Mahmoud et al. [20] studied the strain behavior at the center of tension lap splice length. In the study of Mabrouk and Mounir [21], strain gauges were attached to the middle lap splice and near the end of a lap splice. 

Darwin et al. [22,23] performed a series of experiments on beams with lap-spliced bars of different deformation patterns. They found that the lap splice bond strength can be increased using high relative rib area bars. The relative rib area is the ratio of projected rib area (normal to the bar axis) to the product of the nominal bar perimeter and center-to-center rib spacing. They also considered a number of other variables, e.g., epoxy coating of bars, concrete compressive strength, reinforcement yield strength, coarse aggregate type, lap splice length, and bar deformation pattern. Moreover, they formulated an empirical equation for splice or development length. The recommended the equation of ACI 408 (2003) [24] for bond strength of lap splice was based on the equation developed by Darwin et al. [22]. This equation was further modified based on additional experimental results to derive an expression for lap splices when high-strength concrete was used [25]. 

Esfahani and Rangan [26,27] proposed an equation for lap splice bond strength based on several experimental results. The equation was subsequently modified [28,29] based on the improved understanding of bond strength provided by transverse reinforcement. Esfahani and Kianoush [30] modified the same equation according to studies on the effect of transverse reinforcement on the specimen ductility; they also incorporated the effect of the relative rib area of bars into the equation. 

To formulate a reasonable model for RC structures with lap splices, comprehending the mechanical behavior of rebars and surrounding concrete along the lap splice length is necessary. Because bond or yield failure can occur in the lap splices of RC structures that resist flexural moments, the local strain along lap-spliced rebars can considerably differ between these two failure modes. It is meaningful to measure the strain along lap-spliced rebars and investigate the local behavior of these rebars under the two failure modes.

In the current work, 12 beams were tested; each beam had deformed bars with two lap splices that underwent yield or bond failure under a four-point bending test. By measuring the strain along lap-spliced rebars, a detailed description of the mechanical behavior of these specimens is provided to gain insight on beams with lap splices. Moreover, the beams are compared in terms of ductility, energy dissipation, and bond strength. Finally, the tested bond strengths of the beams are compared with existing bond strength empirical models reported in the literature, i.e., Orangun et al. [2], Darwin et al. [24], and Esfahani and Kianoush [30].

## 2. Specimen Details

Four groups of beams each containing three specimens were tested in this study. For groups 1 and 2, the applied specifications were those of the two beams tested by Darwin et al. [22] (named 2.1 and 4.1 in their report); this enables direct comparison of results. These specimens were intended to undergo bond failure. Groups 3 and 4 were similar to groups 1 and 2, respectively, except that the yield strength of rebars used was lower; these beams were designed to have yield failure. The material properties of these beam groups were similar to those of typical old structures in Korea. 

**Figure 2:**
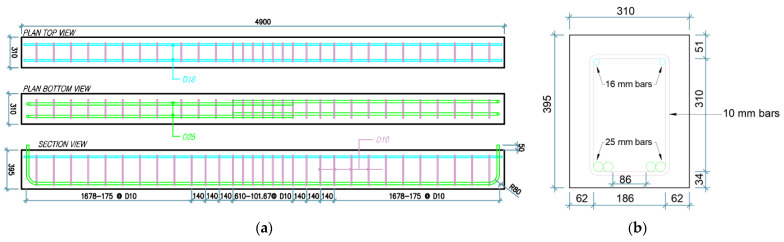
Reinforcement details for C27Y500S10 and C27Y300S10 beams: (**a**) Longitudinal (**b**) Transverse.

**Figure 3:**
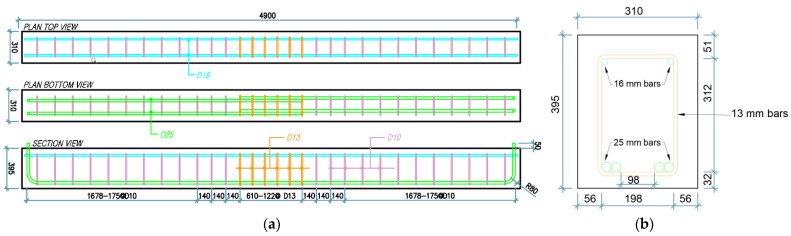
Reinforcement details for C27Y500S13 and C24Y300S13 beams: (**a**) Longitudinal (**b**) Transverse.

The reinforcement details and dimensions of the four beam groups are shown in Figure 2 and Figure 3. All specimens had a cross-section of 310 mm × 395 mm and a simply supported overall span of 4900 mm (4700 mm center-to-center between two roller supports). Two 25 mm diameter tension bars were provided with a lap splice length of 610 mm at the middle of the beam, and two 16 mm diameter continuous compression bars were used for all specimens. Longitudinal bars were confined using 10 mm bars as transverse reinforcement outside the lap splice region. In this lap splice region, groups 1 and 3 had seven 10 mm diameter stirrups at center-to-center spacing of 101.67 mm, and the specimens of groups 2 and 4 were provided with six 13 mm bars for confinement at center-to-center spacing of 122 mm. For groups 1 and 2, the values of concrete compressive strength, $f_{c}^{\prime }$, were 27.1 and 27.5 MPa, respectively, and the yield strength, $f_{y}$, of longitudinal bars and stirrups was 500 MPa. For groups 3 and 4, the values of concrete compressive strength were 26.75 and 23.45 MPa, respectively, and the yield strength of stirrups and spliced longitudinal bars was 300 MPa. Details are summarized in Table 1.

A three-part notation system is used to represent the specimens of each group: two numbers following letter C represent the compressive strength of concrete (in MPa); three numbers following Y represent the yield strength of reinforcement (in MPa); and two numbers following S represent the diameter of stirrups (in mm) in the lap splice region. Specimens C36Y480S10-D and C28Y480S13-D used in the study of Darwin et al. [22] were used as reference in fabrication of our beams. 

In Table 1, $d_{s}$ is the diameter of stirrups; N is the number of stirrups in the lap splice region; d is the beam effective depth; $c_{b}$ and $c_{so}$ are bottom and side covers of longitudinal bars, respectively; $c_{si}$ is half the clear spacing of bars being spliced; $l_{s,test}$ is lap splice length used in the test and $l_{s,ACI}$ is the lap splice length calculated using the equation specified by ACI 318-19 [31].

**Figure 4:**
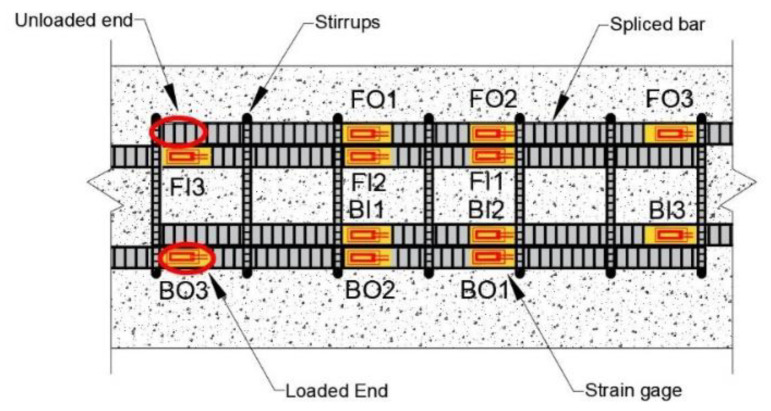
Location of strain gauges for beams with 10 mm diameter stirrups.

**Figure 5:**
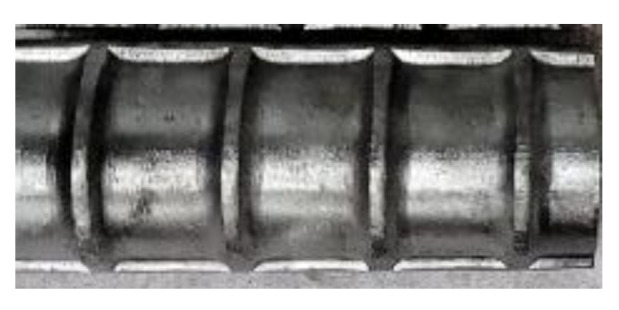
Deformation pattern of longitudinal rebar (Hyundai steel).

Figure 4 shows the location of steel strain gauges with respect to the transverse reinforcement for beams with seven 10 mm diameter stirrups. For beams with six 13 mm diameter stirrups, the strain gauge locations are presented in Section 4.3 of the paper; moreover, the strain behavior of lap splices is discussed. Twelve steel strain gauges were installed on each specimen (three on each bar: two near a quarter point (e.g., FO1 and FO2) of the lap splice length from each end and one at the loaded end (e.g., FO3) of the lap splice), as shown in Figure 4. No strain gauge was installed on the unloaded end of the spliced bar, because bar stresses are not developed at these locations. The location of strain gauges is represented by two letters and a number. The first letters, i.e., F and B, represent the front and back sides of beam specimens, respectively. The second letters, O and I, denote the outer and inner lap-spliced bars, respectively. Numbers 1 and 2 represent positions whose distances are a quarter of the lap splice length; 1 is closer to the unloaded end, and 2 is closer to the loaded end of the lap splice. Number 3 is the loaded end of the lap splice. The deformation pattern of bars used in this study are shown in Figure 5, which is similar to that reported by Darwin et al. [22].

## 3. Test Setup

**Figure 6:**
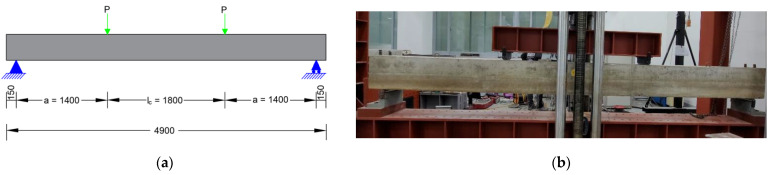
Test setup: (**a**) Schematic of test setup (**b**) Actual test setup.

All specimens were tested using four-point loading tests, as shown in Figure 6. The load was applied to the specimens using a Universal Testing Machine of 200 tonf capacity manufactured by M & T Korea Co., Seoul, Korea. through a spreader steel beam. The length of constant moment, $l_{c}$, was 1800 mm, and the distance between the loading point and nearest support, a, was 1400 mm. The beams were simply supported using steel pedestals at a distance of 150 mm from each end. The load was measured using a load cell at the center of the spreader beam. The midspan deflection of beams was measured using two displacement transducers at the center of the beam. All information (load, displacement, and strain) was recorded on a data logger. Displacement-controlled testing was implemented at a rate of 3.47 mm/min. Cracks were marked on the beams after testing. 

## 4. Test Result and Discussion

### 4.1. Failure Mode

All the specimens in this study were provided with sufficient confinement using transverse reinforcement. Consequently, the controlling variable of specimen failure was the lap splice length. The lap splice length for each group of beams calculated according to the equation specified by ACI 318-19 [31], i.e., $l_{s,ACI}$, is listed in Table 1; $l_{s,ACI}$ is as follows:

(1) $$l_{s,ACI}=\left(\frac{3}{40}\frac{f_{y}}{\lambda \sqrt{f_{c}^{\prime }}}\frac{\Psi_{t}\Psi_{e}\Psi_{s}\Psi_{g}}{\left(\frac{c_{b}+K_{tr}}{d_{b}}\right)}\right)d_{b}$$

where:

(2) $$K_{tr}=\frac{40A_{tr}}{sn}$$

$A_{tr}$ = area of transverse reinforcement crossing the potential plane of splitting (in^2^); *s* = spacing of stirrups (in); *n* = number of bars being spliced or developed; $\Psi_{t}$ is the reinforcement location (casting position) factor = 1 (also known as top bar effect); $\Psi_{e}$ is the coating factor (to account for epoxy coating) = 1; $\Psi_{s}$ is the rebar size (i.e., diameter) factor = 1; $\Psi_{g}$ is the reinforcement grade (yield strength) factor (1 for 300 MPa rebar, and 1.15 for 500 MPa rebar); λ is the lightweight factor = 1; $f_{y}$ and $f_{c}^{\prime }$ are in psi, and $d_{b}$ is in in; $c_{b}\;$is the least of the concrete cover (distance from center of a bar to nearest surface), or one half of the center-to-center spacing of bars; and the confinement term $\left(\frac{c_{b}+K_{tr}}{d_{b}}\right)\;$shall not exceed 2.5. 

**Figure 7:**
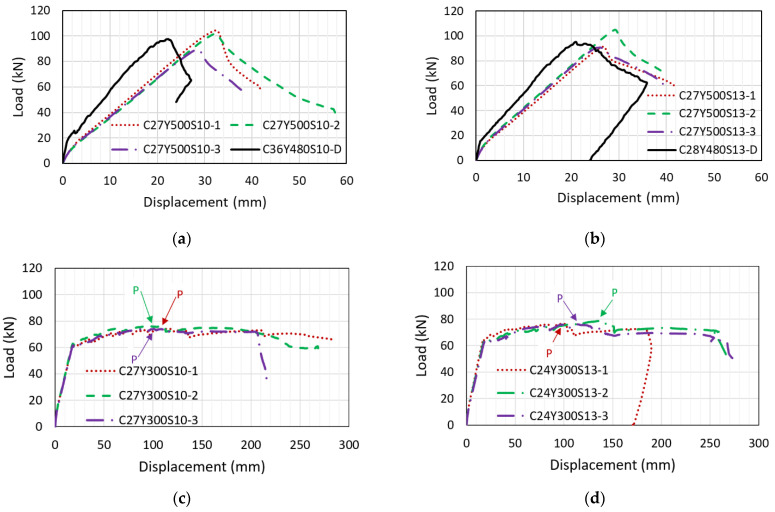
Load–displacement curves: (**a**) C27Y500S10 (**b**) C27Y500S13 (**c**) C27Y300S10 (**d**) C24Y300S13.

Specimens C27Y500S10 and C27Y500S13 were provided with a lap splice length of 610 mm, which is less than that given by $l_{s,ACI}$, indicating that the splice would not provide sufficient bond strength. This explains why these specimens exhibit brittle failure, as indicated by the load–displacement curves shown in Figure 7a,b. Load values increase with increasing displacement, and a sudden drop in the load–displacement curve occurs after reaching a peak value over a small amount of displacement. The foregoing is observed because bond loss occurs between steel and concrete in the lap splice region before the bars yield. The load–displacement curves of reference specimens (C36Y480S10D and C28Y480S13-D) whose specifications were used for groups 1 and 2 are also shown in their corresponding figures. The maximum load and failure behavior of specimens in groups 1 and 2 are similar to Darwin’s specimens (i.e., C36Y480S10 and C28Y480S13, respectively). This means that Darwin’s equation agrees well with our test results.

For C27Y300S10 and C24Y300S13, the lap splice length (610 mm) exceeded that recommended by ACI 318-19 [31]. Therefore, these specimens exhibited sufficient bond strength and ductility. Failure occurred after rebar yielding, and the beams underwent considerable displacement before failure, as indicated by the load–displacement curves shown in Figure 7c,d. The peak load, P, in the load–displacement curves is indicated by arrows in Figure 7c,d.

### 4.2. Cracking Pattern Accordng to Failure Mode

**Figure 8:**
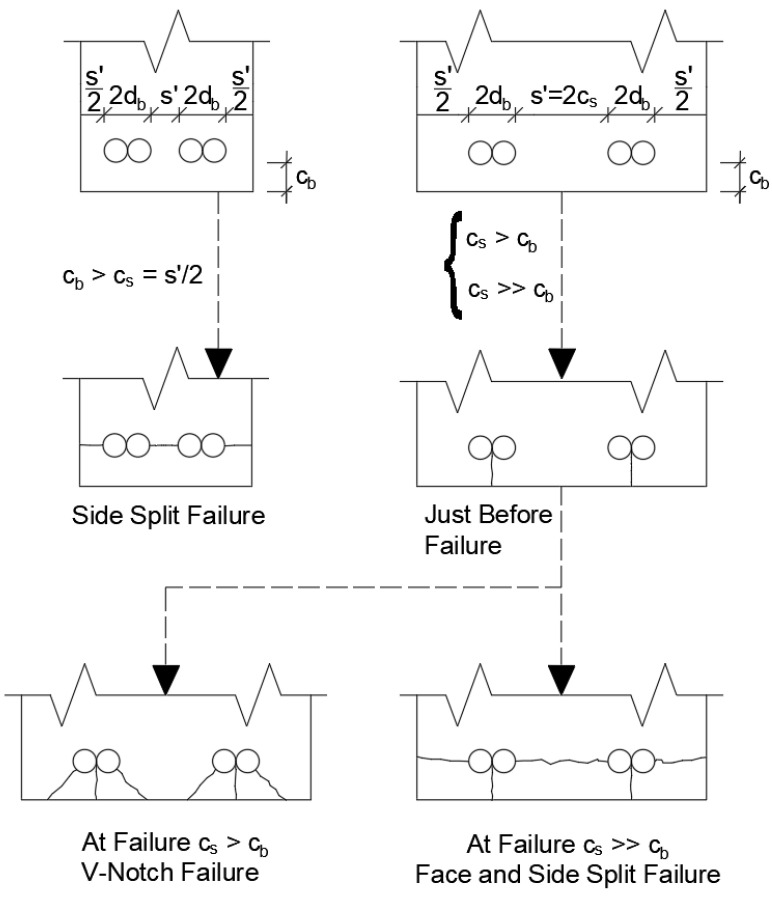
Types of bond failure.

The mode of failure of specimens with lap splices is governed by the cover thickness of lap-spliced bars. Figure 8 shows some basic failure patterns of reinforced concrete members with lap splices [32]. If side cover is less than bottom cover specimen will have side split failure. On the other hand, if bottom cover is less than side cover, depending on the difference between the thickness of these cover either V-Notch failure or face and side split failure may occur.

**Figure 9:**
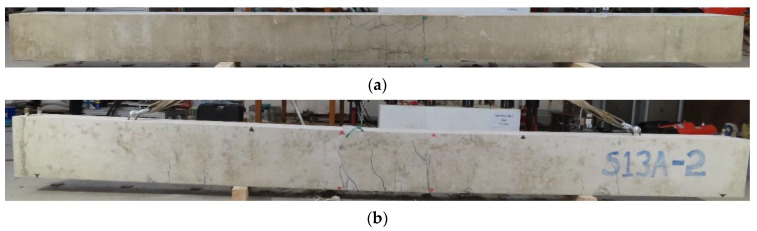
Crack pattern in beam undergoing bond failure (C27Y500S13-2): (**a**) Bottom view (**b**) Side view.

In all our beams, the bottom cover was the controlling factor for the beam failure mechanism rather than the side cover. Figure 9 shows the cracking pattern of bond failure beams C27Y500S10 and C27Y500S13. In our test, significant splitting cracks were observed at the bottom of specimens along the lap splice. Sometimes, minor splitting cracks were observed near the lap splice on the sides of beams. Transverse reinforcement limited the propagation of splitting cracks outside the lap splice region. Significant transverse cracks at the bottom and flexural cracks on the sides of beams occurred at the ends of the lap splice because strain was maximum at these locations along the lap splice length, as shown in Figure 9.

**Figure 10:**
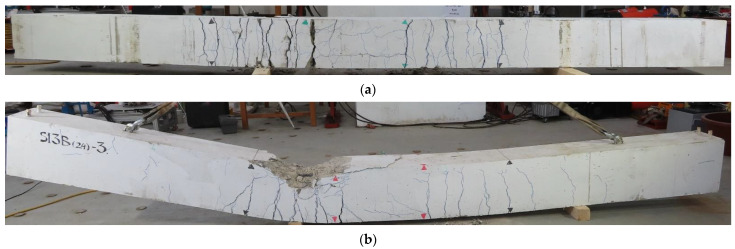
Crack pattern in beam undergoing yield failure (C27Y300S13-3): (**a**) Bottom view (**b**) Side view.

Figure 10 shows the typical cracks on the sides and bottom of specimens (C27Y300S10 and C24Y300S13), which fail due to rebar yielding. Vertical flexural cracks first occurred at the lap splice ends corresponding to a point near the end of the load–displacement curve’s elastic region. Splitting cracks occurred at the sides and bottom of beams near the lap splice. After the peak load (indicated by arrows in Figure 7c,d), the load–displacement curve drops because of concrete crushing in the compression region, as shown in Figure 10b. Because these beams had relatively large bending capacities, severe transverse cracks at the bottom and flexural cracks at the sides occurred outside the lap splice region. The number and width of flexural cracks continued to increase with displacement. The density and width of cracks on one side of the midspan (lap splice) considerably exceeded those of other side cracks because of the uneven load distribution at loading points. This asymmetric cracking and loading behavior were also reflected by the strain in lap splices, as discussed in the following section. 

### 4.3. Strain in Lap-Spliced Rebars

**Figure 11:**
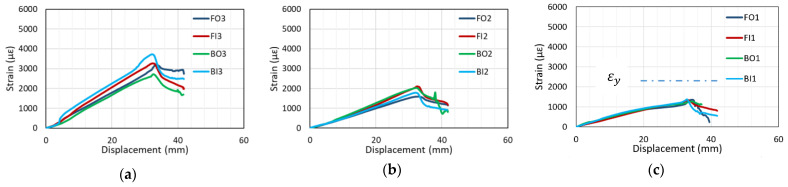
Strain–displacement curves of specimen undergoing bond failure (C27Y500S10-1): (**a**) FO3, FI3, BO3, and BI3 (**b**) FO2, FI2, BO2, and BI2 (**c**) FO1, FI2, BO1, and BI1.

The typical strain–displacement curves of specimens that underwent bond failure (i.e., C27Y500S10 and C27Y500S13) are shown in Figure 11. All strain gauges indicate that the increase in strain is almost linear with increasing displacement. After reaching the peak value at maximum load, the strain decreases; this represents initialtion of bond loss. The strain at the loaded end of the lap splice (FO3, FI3, BO3, and BI3) is maximum, and that at the quarter distance position near the unloaded end of lap splice (FO1, FI1, BO1, and BI1) is minimum. The strain recorded by gauges located at the middle (FO2, FI2, BO2, and BI2) lies in the middle of the strain recorded by the other two gages along the lap-spliced rebar. This shows that the stress at the loaded end of the lap splice is maximum, whereas that at the unloaded end is minimum. This explains why severe transverse cracks are observed at the ends of the lap splice, as shown in Figure 9.

The calculated rebar yield strain, $\varepsilon_{y}$ (i.e., 2272 µ*ε*), based on the design yield strength (500 MPa) of beams that experienced bond failure is shown in Figure 11c. However, the strain at the loaded ends of lap splice is much higher than 2272 µε as shown in Figure 11a, which indicates that the actual yield strength of lap-spliced bars is more than 500 MPa.

**Figure 12:**
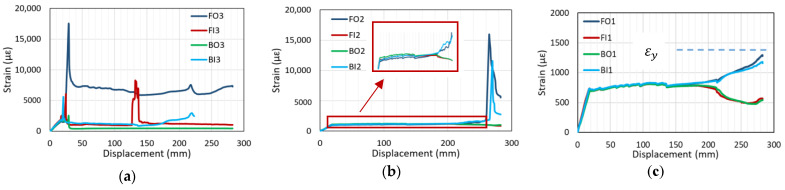
Strain–displacement curves of beam undergoing yield failure (C27Y300S10-1): (**a**) FO3, FI3, BO3, and BI3 (**b**) FO2, FI2, BO2, and BI2 (**c**) FO1, FI2, BO1, and BI1.

**Figure 13:**
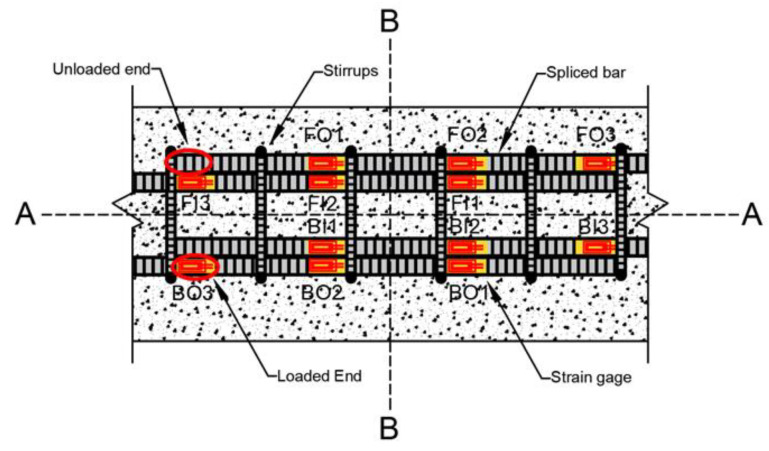
Location of strain gauges for beams with 13-mm diameter stirrups.

The typical strain–displacement curves of specimens that experienced yield failure (i.e., C27Y300S10 and C24Y300S13) are shown in Figure 12. The strain behavior in the elastic region of the load–displacement curve (before yielding) is similar to that of specimens that fail due to bond loss. All gauges indicate a linear increase in strain with displacement. The strain at the loaded end shown in Figure 13 is maximum compared with the strain values at other gauge locations along the lap splice; however, the strain decreases toward the unloaded end (i.e., FO3 > FO2 > FO1). 

The stress flow discontinuity in the lap-spliced rebar causes stress concentration at the loaded ends (FO3, FI3, BO3, and BI3); this explains why maximum strain is attained at these locations. If the strain exceeds the elastic limit, rebar yielding occurs; this corresponds to a spike in the strain-displacement curve when the displacement is 20 mm, as shown in Figure 12a. The increasing load generates cracks at these locations, consequently redistributing the rebar stress. This redistribution releases the high strain at the loaded end. Consequently, the strain drops to the plastic strain and remains almost constant with increasing displacement. The strain at quarter length positions along the lap splice (FO2, BO2, FO1, BO1, etc.) slightly increases until the maximum load is attained; at this instance, the displacement reaches 110 mm.

After the beam attains its maximum load capacity (as indicated by the arrows in Figure 7c,d, concrete in the compression region is crushed, as illustrated in Figure 10b; consequently, all load is borne by tension bars. Due to this sudden transfer of load from compression to tension region there is a second spike in the strain–displacement curve of strain gauge FI3, as shown in Figure 12a, corresponding to the displacement of 120 mm. Because the compressive crushing of concrete is asymmetric with respect to the midspan as shown in Figure 10b, strain curve separation occurs, as shown in Figure 12a–c. With the occurrence of concrete crushing on one side (FO3, FO2, FO1, BI3, BI2, and BI1), strain starts to increase, whereas on the other side with relatively small cracks (FI3, FI2, FI1, BO3, BO2, and BO1), strain starts to decrease. This strain curve separation increases with the asymmetric increase in cracks. With increasing strain curve separation, the strain at the quarter length positions near the loaded end of lap splice (BI2 and FO2) spikes, indicating rebar yielding, as shown in Figure 12b. The magnified view of strain values recorded by the middle strain gauge is also shown in Figure 12b.

The calculated yield strain, $\varepsilon_{y}$ (i.e., 1363 µε), based on the design yield strength (300 MPa) of beams that undergo yield failure is shown in Figure 12c. However, the actual yield strength, $f_{y}$, of lap-spliced beams was found to exceed the design yield strength.

Figure 13 shows the location of strain gauges for beams with 13 mm diameter stirrups. Similar to the strain–displacement curves shown in Figure 12, the behavior of rebars on the same side starting from Centerline A of the lap splice exhibits the same trends (e.g., the strain curves of FO3, FO2, and FO1 have same trends as those of BI3, BI2, and BI1). However, strain values probably differ because of the asymmetric construction of beams with respect to Centerline A. Although FO3 and BI3 are both located at the loaded end and on the same side from Centerline B, the strain readings in these two gauges differ because of the unsymmetric loading condition of the test setup with respect to Centerline B. If asymmetric behavior about Centerline A and B becomes larger, local stress concentration in lap-splice bonding will be severe and then strength and stiffness of the beam will be reduced accordingly.

### 4.4. Initial Stiffness, K

The calculated initial stiffness (slope of load–displacement curve in the linear region), K, is listed in Table 2. Specimens that experienced bond failure have relatively high initial stiffness than specimens that underwent yield failure. Because the actual compressive strength of tested specimens was less than that of Darwin’s specimens [22] (C36Y480S10-D and C28Y480S13-D), the latter samples had greater initial stiffness than the tested specimens listed in Table 2. 

### 4.5. Ductility, µ

The ductility, µ, of specimens (summarized in Table 2) is calculated using the method proposed by Cohn and Bartlett [24], as shown in Figure 1. The ductility of specimens that exhibited yield failure was approximately 10 times greater than those of specimens that underwent bond failure. This shows that the prevention of bond failure in flexural reinforced concrete members is important in seismic design. 

### 4.6. Energy Dissipation, E

The area under the load–displacement curve that accounts for the energy dissipation, E, is an important factor to understand the seismic behavior of specimens. The higher the energy dissipation, the better the seismic performance of a structural member. The energy dissipation up to 85% of the maximum load on post peak portion of load-displacement curves in all specimens is summarized in Table 2. In this study, specimens that exhibited yield failure had approximately 8.5 times greater energy dissipation than those that underwent bond failure.

### 4.7. Bond Strength

The experimental bond strength, $u_{test}$, is given by the following:

(3) $$u_{test}=\frac{A_{b}f_{s}}{\pi d_{b}l_{d}}$$

where $d_{b}$ and $A_{b}$ represent the diameter and area of a longitudinal rebar, respectively; $l_{d}$ is the development or splice length; $f_{s}\;$is the failure stress of steel calculated by elastic cracked section analysis of beams considering a single continuous tension bar using the following equation:

(4) $$f_{s}=\frac{M_{test}}{A_{s}jd}\;$$

(5) $$M_{test}=\frac{Pa}{2}\;$$

where $M_{test}$ is the maximum moment at failure; P is the maximum load from bending test, presented in Table 3; a is the distance between support and nearest loading point, as shown in Figure 6a; $A_{s}$ is the area of steel rebars, and jd is the moment arm (from center of steel to center of concrete in compression) calculated by assuming the elastic behavior of a section in a reinforced concrete beam without rebar lap splices. The calculation of each perimeter is summarized in Table 3. In this table, ρ is the reinforcement ratio given by following equation:

(6) $$\rho =\frac{A_{s}}{bd}\;$$

where d is the effective depth, and b is the width of beam. The calculation of$\;f_{s}$ is also summarized in Table 3, where $E_{s}$ and $E_{c}\;$are the elastic moduli of steel and concrete, respectively, and *n* is the modular ratio.

Orangun et al. [2] proposed the following relationship for the bond strength of a lap splice:

(7) $$u=\left[1.2+3\left(\frac{c}{d_{b}}\right)+50\left(\frac{d_{b}}{l_{s}}\right)+k_{tr}^{\prime }\right]\sqrt{f_{c}^{\prime }}\;\mathrm{for}\;\frac{c}{d_{b}}\;\leq \;2.5$$

where,

(8) $$k_{tr}^{\prime }=\frac{A_{tr}f_{yt}}{500\;s\;n}\;\mathrm{for}\;k_{tr}^{\prime }\;\leq \;3.0$$

*u* = theoretical bond stress (psi); *c* = the smaller between $c_{b}$ and $c_{s}$; $c_{b}\;$= clear (bottom or side) cover to main reinforcement (in); $c_{s}\;$= half the clear spacing of bars or splices, or half the available concrete width per bar or splice resisting splitting in the failure plane (in); $d_{b}\;$= diameter of reinforcing bar (in); $l_{s}$ = lap splice length (in); $f_{c}^{\prime }\;$= compressive strength of concrete (psi); $k_{tr}^{\prime }$ = confinement factor (no unit); and $A_{tr}$ = area of transverse reinforcement crossing the potential plane of splitting adjacent to a single-anchored reinforcing bar (in^2^).

Darwin et al. [22] suggested the following equation for bond strength:

(9) $$u=\frac{{\left(f_{c}^{\prime }\right)}^{\frac{1}{4}}}{\pi d_{b}l_{d}}\left[63l_{d}\left(c_{m}+0.5d_{b}\right)+2130A_{b}\right]\left(0.1\frac{c_{M}}{c_{m}}+0.9\right)+2226t_{r}t_{d}\frac{NA_{tr}}{n}\;$$

where $l_{d}$ = development or splice length (in); $d_{b}$ = nominal bar diameter (in); $A_{b}$ = bar area (in^2^); $f_{c}^{\prime }$ = concrete compressive strength (psi); $c_{M}$ = maximum value of $c_{s}$ or $c_{b}$ (in); $c_{m}$ = minimum value of $c_{s}$ or $c_{b}$ (in); $c_{M}$ and *C_m_* must satisfy $\frac{c_{M}}{c_{m}}$ ≤ 3.5; $c_{s}$ = min ($c_{si}$ + 0.25 in, $c_{so}$) or min ($c_{si}$, $c_{so}$) (in); $c_{si}$ = one half the clear spacing of bars (in); $c_{so}$ = side cover of reinforcing bars (in); $c_{b\;}$= bottom cover of reinforcing bars (in); $t_{r}$ = 9.6$R_{r}$ + 0.28 (taken as 1); $t_{d}$ = 0.72$d_{b}$ + 0.28; $R_{r}$ = relative rib area; $A_{tr}$ = area of each stirrup or tie crossing the potential plane of splitting adjacent to the reinforcement being developed or spliced (in²); s = spacing of transverse reinforcement (in); n = number of bars being developed or spliced along the plane of splitting.

The bond strength equation proposed by Esfahani and Kianoush 2005 [30] is as follows:

(10) u=uc1+1M1.85+0.024M(0.88+0.12CmedC)×(1+0.015fRAtAbCs) 

where,

(11) $$M=cosh\left(0.0022l_{d}\sqrt{3\frac{f_{c}^{\prime }}{d_{b}}}\right)\;$$

and

(12) $$u_{c}=2.7\frac{\frac{C}{d_{b}}+0.5}{\frac{C}{d_{b}}+3.6}\;\sqrt{f_{c}^{\prime }}\;$$

In the above equations, *C* = minimum of bottom cover, side cover, or half the spacing of bars (mm); $C_{med}\;$= median of bottom cover, side cover, or half the spacing of bars (mm); *s* = stirrup spacing (mm); $l_{d}$ = development or splice length (mm); $f_{c}^{\prime }\;$= compressive strength of concrete (MPa); $d_{b}$ = bar diameter (mm); $A_{t}$ = area of single transverse reinforcement (mm²); $A_{b}$ = area of single longitudinal tension bar (mm²); $f_{R}$ = 1 ($f_{R}$ = 1 if $R_{r}$ < 0.11, and $f_{R}$ = 1.6 if $R_{r}$ ≥ 0.11). 

For specimens that exhibited bond failure (i.e., two specimens tested by Darwin et al. [22] and six specimens tested in this study), the ratio of bond strengths calculated by the equations above to the bond strengths measured by corresponding tests are summarized in Table 4; their mean values and standard deviations (SDs) are also listed in this table. The foregoing equations satisfactorily predict the bond strength of specimens. Equation proposed by Orangun et al. [2] yields the best prediction with a mean value of 1.04 and an SD value of 0.09. 

For specimens that underwent yield failure, the equation of Esfahani and Rangan [30] most accurately predicts the bond stress among the three equations, listed in Table 5. For the listed specimens, $f_{y}\;$instead of $f_{s}$ is used to calculate bond stress at yielding, $u_{test}^{y}$, in Equation (3). For this equation, $f_{y}$ is calculated from the measured strain of four lap-spliced bars at the starting point of the first spike in the strain–displacement curves shown in Figure 12a; the minimum value is used to calculate $u_{test}$ in specimens. 

## 5. Conclusions

In this study, two lap-spliced beams tested by Darwin et al. [22] were used as reference to fabricate four groups of specimens with the same dimensions and lap splice length. Several strain gauges were attached to the beam rebars to study the local behavior of lap splices that undergo yield or bond failure; all specimens were tested under four-point loading. The following conclusions were obtained:

1. As supported by previous literature, strain gauge data shows that strain is maximum at the loaded end of a lap splice and progressively decreases toward the unloaded end.
2. Beams without sufficient lap splice length fail in a brittle manner (bond failure). For these specimens, the increase in strain with displacement at all gauge points is almost linear. Then, after reaching a peak value, the strain drops, indicating the initiation of bond loss.
3. Beams with sufficient lap splice length fail in a ductile manner (yield failure). For these beams, rebar yielding occurs at the loaded end of the lap splice and causes stress redistribution. Concrete in the compression region is crushed on one side from midspan after reaching the peak load. This remarkably increases the asymmetric behavior of beam specimens such that bar strains increase at the crushed section but decrease on the opposite side of midspan. Yield failure specimens as compared to bond failure beams exhibit approximately 10 and 8.5 times greater ductility and energy dissipation, respectively.
4. The bond strengths of our specimens, which undergo bond failure, are compared with the bond strength values yielded by the three empirical equations proposed by Orangun et al. (1977) [2], Darwin et al. (1995) [22], and Esfahani and Kianoush (2005) [30]. The comparison indicates that these equations yield a satisfactory prediction of results. In particular, equation suggested by Orangun et al. [2] yields the best prediction.

## Figures and Tables

**Table 1:** Specimen details.

Specimens	Notation	$f_{c}^{\prime }$ MPa	$f_{y}$ MPa	$d_{s}$ mm	N	d mm	$c_{so}$ mm	$c_{si}$ mm	$c_{b}$ mm	$l_{s,test}$ mm	$l_{s,ACI}$ mm
Group 1	C27Y500S10	27.10	500	10	7	348.50	62.00	43.00	34.00	610	1014
Group 2	C27Y500S13	27.50	500	13	6	350.50	56.00	49.00	32.00	610	1006
Group 3	C27Y300S10	26.75	300	10	7	348.50	62.00	43.00	34.00	610	532
Group 4	C24Y300S13	23.45	300	13	6	350.50	56.00	49.00	32.00	610	569
2.1 [22]	C36Y480S10-D	36.19	480	10	7	347.98	57.15	43.33	33.73	610	847
4.1 [22]	C28Y480S13-D	28.19	480	13	6	348.49	52.40	48.92	31.75	610	959

**Table 2:** Initial stiffness, ductility, and energy dissipation.

Specimen	Failure Mode	$f_{c}^{\prime }$ MPa	K kN/mm	µ	E kN∙mm
C27Y500S10-1	Bond (Brittle) Failure	27.1	3.2211	1.11	2100
C27Y500S10-2			3.1241	1.15	2132
C27Y500S10-3			3.1063	1.15	1670
C27Y500S13-1		27.5	3.3712	1.17	1640
C27Y500S13-2			3.5299	1.14	1975
C27Y500S13-3			3.5018	1.32	1867
C27Y300S10-1	Yield (Ductile) Failure	26.75	3.3195	13.43	19,228
C27Y300S10-2			3.3868	10.92	16,097
C27Y300S10-3			3.2947	9.82	14,117
C24Y300S13-1		23.45	3.9458	9.81	12,877
C24Y300S13-2			3.6011	12.13	18,068
C24Y300S13-3			3.3293	11.60	17,015
C36Y480S10-D	Bond (Brittle)	36.2	4.2122	1.23	1589
C28Y480S13-D	Failure	28.2	4.2433	1.38	1772

**Table 3:** Loads at failure and calculated stresses of rebar at failure.

Specimen	*P* kN	$f_{c}^{\prime }$ MPa	*ρ*	$E_{c}$ MPa	*n*	*k*	*j = 1 − k/3*	$f_{s}$ MPa
C27Y500S10-1	104.23	27.1	0.009083	24,467.10	8.99	0.41	0.86	494.71
C27Y500S10-2	101.83	27.1	0.009083	24,467.10	8.99	0.41	0.86	483.32
C27Y500S10-3	89.32	27.1	0.009083	24,467.10	8.99	0.41	0.86	423.94
C27Y500S13-1	91.62	27.5	0.009031	24,647.01	8.93	0.41	0.86	431.93
C27Y500S13-2	104.87	27.5	0.009031	24,647.01	8.93	0.41	0.86	494.37
C27Y500S13-3	90.64	27.5	0.009031	24,647.01	8.93	0.41	0.86	427.31
C27Y300S10-1	74.26	26.75	0.009083	24,308.59	9.05	0.41	0.86	352.65
C27Y300S10-2	76.27	26.75	0.009083	24,308.59	9.05	0.41	0.86	362.20
C27Y300S10-3	74.02	26.75	0.009083	24,308.59	9.05	0.41	0.86	351.49
C24Y300S13-1	76.47	23.45	0.009031	22,759.84	9.67	0.43	0.86	362.92
C24Y300S13-2	78.78	23.45	0.009031	22,759.84	9.67	0.43	0.86	376.80
C24Y300S13-3	76.03	23.45	0.009031	22,759.84	9.67	0.43	0.86	363.09
C36Y500S10-D	98.39	36.2	0.009096	28,278.22	7.78	0.38	0.87	453.27
C28Y500S13-D	98.08	28.2	0.009083	24,958.73	8.81	0.41	0.86	455.48

k=2nρ(nρ)2, $E_{s}$ = 220,000 MPa, $E_{c}=4700\sqrt{f_{c}^{\prime }}$, n=Es Ec, $A_{S}$ = 981.25 mm^2^

**Table 4:** Comparison of bond strengths of lap splices in beam specimens.

**Specimen**	$u_{test}$ **MPa**	$u_{darwin}$ MPa	$u_{orangun}$ MPa	$u_{esfahani}$ MPa	$u_{test}/$ $u_{darwin}$	$u_{test}/$ $u_{orangun}$	$u_{test}/$ $u_{esfahani}$
C27Y500S10-1	5.07	4.05	4.46	3.92	1.27	1.14	1.29
C27Y500S10-2	4.95	4.05	4.46	3.92	1.24	1.11	1.26
C27Y500S10-3	4.34	4.05	4.46	3.92	1.09	0.97	1.11
C27Y500S13-1	4.43	4.36	4.39	4.22	1.02	1.01	1.05
C27Y500S13-2	5.07	4.36	4.39	4.22	1.16	1.15	1.20
C27Y500S13-3	4.38	4.36	4.39	4.22	1.00	1.00	1.04
C35Y500S10-D	4.64	4.26	5.14	4.52	1.09	0.90	1.03
C27Y500S13-D	4.67	4.29	4.44	4.45	1.09	1.05	1.05
Mean					1.12	1.04	1.13
SD					0.10	0.09	0.11

**Table 5:** Comparison of bond stress for yield failure of beam specimens.

**Specimen**	$f_{y}$ MPa	$u_{test}^{y}$ MPa	$u_{darwin}$ MPa	$u_{orangun}$ MPa	$u_{esfahani}$ MPa	$u_{test}^{y}/$ $u_{darwin}$	$u_{test}^{y}/$ $u_{orangun}$	$u_{test}^{y}/$ $u_{esfahani}$
C27Y300S10-1	335	3.43	4.04	4.44	3.90	0.86	0.77	0.88
C27Y300S10-2	384	3.93	4.04	4.44	3.90	0.99	0.89	1.01
C27Y300S10-3	365	3.74	4.04	4.44	3.90	0.94	0.84	0.96
C24Y300S13-1	363	3.72	4.19	4.06	4.03	0.89	0.92	0.92
C24Y300S13-2	344	3.52	4.19	4.06	4.03	0.84	0.87	0.88
C24Y300S13-3	395	4.05	4.19	4.06	4.03	0.97	1.00	1.00
Mean						0.91	0.88	0.94
SD						0.06	0.07	0.06

## Data Availability

Data sharing is not applicable to this article.
